# Intrabody B1 targeting TDP-43 modulates neuroinflammatory and metabolic pathways in a preclinical ALS model

**DOI:** 10.1016/j.neurot.2026.e01032

**Published:** 2026-08-06

**Authors:** Y. Al Ojaimi, A. Dupuis, M. Palla, D. Lanznaster, R. Hergesheimer, L. Galineau, S. Serriere, A.A. Chami, P. Martineau, T. Alouane, S. Marouillat, C. Dupuy, B.L. Schneider, C. Veyrat-Durebex, F. Masse, F. Trovero, C.R. Andres, P. Corcia, P. Vourcʼh, H. Blasco

**Affiliations:** a Université de Tours, INSERM, Imaging Brain & Neuropsychiatry IBraiN U1253, Tours, France; b CHU de Tours, Service de Biochimie et Biologie Moléculaire, Tours, France; c Plateforme Imagerie Préclinique, US61 ASB, Université de Tours, CHU Tours, Inserm, Tours, France; d IRCM, Univ Montpellier, Inserm, ICM, Montpellier, France; e CHU de Tours, Unité Transversale des Technologies Innovantes de Laboratoire (UTTIL), Tours, France; f Plateforme de Génomique, US61 ASB, Université de Tours, CHRU Tours, INSERM, Tours, France; g Plateforme de Métabolomique et D'Analyses Chimiques, US61 ASB, Université de Tours, CHRU Tours, INSERM, 37032 Tours, France; h Bertarelli Platform for Gene Therapy, Swiss Federal Institute of Technology (EPFL), Lausanne, Switzerland; i Key-Obs, Orléans, France; j CHU de Tours, Service de Neurologie, Tours, France

**Keywords:** TDP-43, scFv, AAV-Derived therapeutic, Omics, Proteinopathy

## Abstract

TDP-43 pathology is a hallmark of Amyotrophic Lateral Sclerosis (ALS), yet no therapeutic strategy effectively targets its upstream molecular consequences. Here, we investigated whether the anti-TDP-43 intrabody scFv B1 modulates neuroinflammatory and metabolic pathways in a preclinical ALS model, and whether these effects translate into functional benefit after symptom onset. Using phage display, we previously identified single-chain variable fragments (scFvs) binding TDP-43, including the candidate therapeutic scFv B1. In NSC-34 motor neuron-like cells overexpressing human wildtype TDP-43, B1 reduced NF-κB activation, consistent with disruption of TDP-43–driven inflammatory signaling. For *in vivo* assessment, B1 was delivered via AAV-CAP.B10 after symptom onset in the hTDP-43(WTxA315T) transgenic mouse model, enabling neuro-specific expression. Two cohorts were analyzed - longitudinal (nine months) and terminal (six months post-treatment) - through behavioral testing, PET imaging, metabolomics, transcriptomics, and plasma biomarker analyses. B1 achieved robust CNS expression and modulated several disease-relevant molecular pathways. RNA-sequencing revealed attenuation of NF-κB–related inflammatory signatures and partial normalization of metabolic and trophic gene expression. Metabolomic profiling identified shifts toward wild-type-like levels in oxidative stress, mitochondrial, and membrane phospholipid metabolites. Despite these molecular effects, symptomatic B1 administration did not improve motor behavior or reduce plasma neurofilament light chain (NfL) concentrations. Notably, plasma TDP-43 levels were stabilized, indicating systemic target engagement. Collectively, scFv B1 modulates upstream pathogenic processes associated with TDP-43 proteinopathy but is insufficient to reverse established neurodegeneration after symptom onset, underscoring the need for earlier and likely combinatorial intervention strategies in ALS.

## Introduction

Amyotrophic Lateral Sclerosis (ALS) is a devastating neurodegenerative disease characterized by the progressive and selective loss of upper and lower motor neurons. The disease typically manifests muscle weakness, spasticity, and atrophy, ultimately leading to paralysis and respiratory failure within a few years of symptom onset. Despite decades of research, treatment options remain extremely limited, and current therapies provide only modest effects on disease progression and patient survival. This urgent medical need highlights the importance of developing innovative therapeutic strategies that directly target the molecular mechanisms underlying ALS [1,2].

One of the most consistent pathological features of ALS is the mislocalization and aggregation of the transactive response DNA-binding protein 43 kDa (TDP-43). In the majority of ALS cases, TDP-43 loses its normal nuclear localization and accumulates in the cytoplasm, where it forms insoluble inclusions. This disruption contributes both to a loss of physiological nuclear functions and a gain of toxic cytoplasmic functions. Consequently, therapeutic strategies aiming to prevent or reverse TDP-43 pathology are of particular interest [3].

Antibody-based therapies have transformed treatment paradigms in several diseases. Single-chain variable fragments (scFvs) are antibody fragments composed of the variable region of heavy and light chains connected by a flexible linker. When expressed intracellularly, they function as intrabodies, directly binding to disease-related proteins such as TDP-43 within neurons [4].

However, ALS is not a disease driven solely by TDP-43 pathology. Other processes, including neuroinflammation, metabolic alterations, mitochondrial dysfunction, and altered RNA metabolism, play key roles in disease progression. It is therefore crucial to evaluate candidate interventions not only for their direct effect on TDP-43, but also for their impact on these broader pathological processes. Indeed, targeting TDP-43 pathology is biologically compelling, yet target engagement alone does not necessarily restore the multiple disrupted pathways underlying neurodegeneration. Because validated response biomarkers in ALS remain scarce, with neurofilament light chain (NfL) as the most established biomarker, there is a critical need for integrated readouts that connect molecular intervention to functional and clinical correlates. A multimodal strategy combining *in vitro* and *in vivo* systems with central and peripheral analyses, multi-omics profiling, and advanced brain imaging is therefore essential to capture the full impact of therapeutic intervention. Recent studies have highlighted a strong relationship between TDP-43 pathology and energetic metabolism and neuroinflammation, providing the scientific rationale for focusing on these readouts in the present study [[5], [6], [7], [8]].

In this study, we report on the preclinical evaluation of an scFv intrabody targeting TDP-43, with a particular focus on its capacity to modulate neuroinflammatory and metabolic pathways. Using a phage-display screening approach against recombinant full-length wild-type TDP-43, we previously identified four TDP-43-specific scFv clones (B1, A2, B6, and D7), of which only B1 and D7 were successfully expressed and shown to bind wtTDP-43 by ELISA and cellular colocalization [4]. *In silico* epitope prediction further indicated that B1 targets the RRM1 domain and/or the C-terminal end of the prion-like domain, two regions central to TDP-43 aggregation. Notably, B1 was the only clone capable of normalizing TDP-43-induced NF-κB activation and partially restoring TDP-43-associated metabolic disturbances, providing the rationale for its selection for further characterization in the present study. In this study, we first tested B1 in NSC-34 cells overexpressing TDP-43 to assess its impact on downstream signaling pathways. To assess its therapeutic potential *in vivo*, we delivered AAV-CAP.B10–encoded B1 after symptom onset. This vector, previously used to express a TDP-43–targeting scFv, enables robust neuronal expression [9]. Two cohorts were analyzed: a longitudinal cohort monitored for nine months after treatment and a terminal cohort examined six months post-injection, allowing a multi-modal assessment of both central and peripheral effects, including behaviour, PET imaging, metabolomics, transcriptomics, and plasma biomarkers. By integrating these complementary readouts, we aim to determine whether molecular target engagement translates into functional benefit when treatment is initiated at a symptomatic stage, and we highlight the importance of a multidimensional approach to therapeutic evaluation in ALS.

## Materials and methods

### Cell culture and transfection

NSC-34 cells (ATCC) were the main cell line of choice. This hybrid cell line is derived from the fusion of mouse spinal cord motor neurons and neuroblastoma cells, giving them properties similar to motor neurons. Cells were maintained in Dulbecco's Modified Eagle's Medium (DMEM) supplemented with 5% (v/v) fetal bovine serum (FBS) at 37 °C in an incubator maintaining an atmosphere of 5% CO_2_. Cells were split once they reached 90% confluency.

Six-well plate (CytoOne) were seeded with 0.3x10^6^ cells par well in DMEM/5%FBS. Once the cells reached 60% confluency, they were transfected for 48h using JetPEI transfection reagent (Polyplus transfection) following the manufacturer's protocol. A total of 4.5 μg of plasmids were always used. For the TDP-43 condition, 3 μg of wt-TDP-43-expressing plasmid with 1.5 μg of empty plasmid (pcDNA3.3-TOPO) was used. For the TDP-43/scFv co-transfections, the empty plasmid was replaced with 1.5 μg of scFv-expressing plasmid. Plasmids expressing 4 different anti-TDP-43 scFvs were tested at this stage (A2, B1, B6, D7) along with the plasmid expressing the control scFv 13R4 which does not target TDP-43 [4].

### TDP-43 and p65 expression

Following 48 h of overexpression in NSC-34 cells, the cells were lysed using ice-cold lysis buffer (PBS pH 8.0, 1% Triton-X 100, 100 mM MgCl2, 1 mM DTT) supplemented with 1X Halt™ Protease and Phosphatase Inhibitor Cocktail (Thermo Fisher Scientific) and Pierce™ Universal Nuclease (Thermo Fisher Scientific). After a 30 min incubation on ice, the lysates were centrifuged at 900×*g* to remove debris. The resulting supernatant, representing the total protein fraction, was collected, and protein concentration was determined using the Pierce™ 660 nm Protein Assay Reagent (Thermo Fisher Scientific).

For western blot analysis, 20 μg of proteins per sample was resolved on an SDS-PAGE gel, transferred onto a 0.2 μm PVDF membrane, and blocked in 5% fat-free milk dissolved in TBS-Tween20 (0.1%) for 2 h at room temperature. Membranes were then incubated overnight at 4 °C with either a rabbit anti-TDP-43 antibody (12892-1-AP, Proteintech, dilution 1:5000), a mouse anti- NF-κB p65 antibody (MAB3026, Sigma Aldrich, dilution 1:1000), or an HRP-conjugated anti-6∗His antibody (HRP-66005, Proteintech, dilution 1:100,000). Following three 10-min washes in TBST, membranes were incubated with the corresponding HRP-conjugated secondary antibody (anti-rabbit #W4011 or anti-mouse #W4021, Promega, dilution 1:10,000) for 1 h at room temperature. Protein bands were visualized using ECL substrate (Bio-Rad) and normalized with ImageLab software relative to the stain-free total protein.

### Animal groups

For this study, we used the GemPharmatech hTDP-43(WTxA315T) transgenic mouse model (C57BL/6JGpt-Tg (hTARDBP A315T, hTARDBP)9/Gpt; strain #T054620) and wildtype mice with the same genetic background. Manufacturer validation data (GemPharmatech) confirm TDP-43 overexpression, inclusion formation, and a neuroinflammatory profile comparable to SOD1 transgenic mice, with insoluble TDP-43 accumulation primarily restricted to the spinal cord in this mice model.

All experimental cohorts comprised exclusively female mice. This decision was made deliberately based on our pre-clinical characterization of both the TDP-43 JAX and TDP-43 GPT lines: male mice develop motor deficits significantly more rapidly and have markedly shorter survival times, making longitudinal therapeutic evaluation impractical in male subjects. By using exclusively female mice, we ensured minimal attrition during the study period and maximised the reliability of longitudinal comparisons across cohorts.

We included three groups of mice (20 animals per group): wild-type (WT) mice injected with saline, transgenic (Tg) mice injected with the AAV vector expressing a control scFv (13R4, which does not target TDP-43), and Tg mice treated with the AAV expressing the TDP-43-specfic scFv B1. All injections were performed after symptom onset and were administered intravenously at baseline at a dose of 6 × 10^11^ vector genomes (vg) per mouse. Two complementary experimental cohorts were used.

The longitudinal cohort was designed for peripheral biomarker analyses and phenotypic follow-up. Animals were monitored until 9 months post-treatment. Body weight was measured weekly, while motor function was assessed longitudinally using wire maneuver, grip strength, tail position and gait analysis every 8 weeks, and rotarod testing every 16 weeks. For the gait impairment, an arbitrary qualitative score system was used: score 0: no motor impairment, score 1 (tremor while walking), score 2 (lowered pelvis and swimming gait while moving forward), score 3 (struggling to move forward and dragging its abdomen on the ground), and score 4 (euthanasia endpoint in which the mouse failed to upright itself within 30 s). A similar score system was applied for the tail position: score 0 (tail continuously dragging), score 0.5 (tail intermittently dragging), score 1 (tail parallel to the ground) and score 2 (tail slightly elevated). Peripheral samples, including plasma and dried blood spots (DBS), were collected about every 16 weeks for biomarker and metabolomics analyses; prior to treatment initiation (t0), 4 months post-treatment (t1), and 9 months post-treatment (t2).

A second, terminal cohort was sacrificed 6 months post-treatment for tissue-based analyses. Animals underwent ^18^FDG and ^18^F-DPA-714 PET imaging prior to sacrifice. Plasma was collected, and mice were euthanized with Dolethal followed by intracardiac PBS perfusion. Brain and spinal cord were harvested, with brain tissue processed for histological and biochemical analyses and all tissues stored at −80 °C.

A schematic overview of the *in vivo* protocol is provided in Fig. 1.

**Fig. 1:**
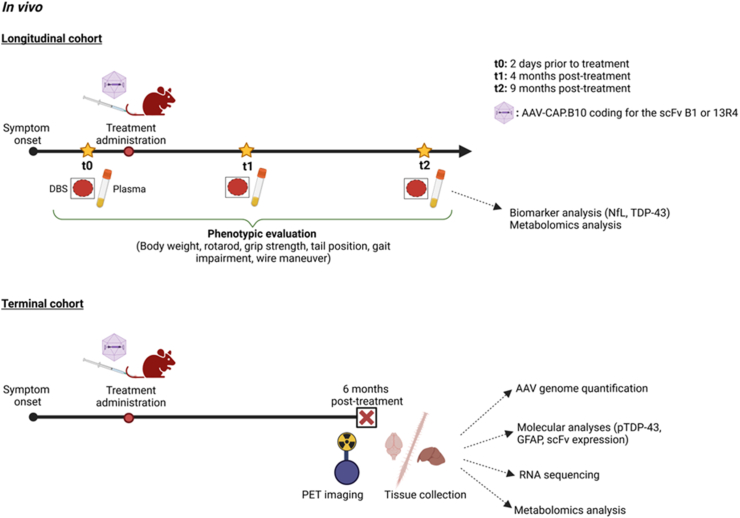
Representative scheme of the *in vivo* experiments.

### Quantification of viral DNA

Genomic DNA was isolated from 3 mg of lyophilized brain, spinal cord or liver tissue using the Maxwell® RSC Viral Total Nucleic Acid Purification Kit (AS1330, Promega) according to the manufacturer's instructions. For each assay, 40 ng of total DNA was used as input for droplet digital PCR (ddPCR). Duplex ddPCR assays were performed using the Bio-Rad probe-based assays: *KIF21A* (HEX probe) as the endogenous reference and the AAV polyA sequence (FAM probe) as the target. A reaction containing nuclease-free water and ddPCR supermix was included as a negative control. Droplet generation, PCR amplification and droplet reading were performed using Bio-Rad ddPCR instrumentation and data were analyzed with the QX Manager software (Bio-Rad). Absolute copy numbers for each assay were obtained after Poisson correction by the QX software. Results are reported as AAV genome copies per diploid genome by normalizing AAV copies to *KIF21A* readout, assuming *Kif21A* is present at two copies per diploid genome.

### Immunofluorescence on NSC-34 cells and brain sections

For immunofluorescence on NSC-34 cells, cells were seeded at a density of 10,000 cells per well in 8-well chamber slides coated with 0.1 mg/mL poly-D-lysine (Gibco™). After 48 h of expression, cells were fixed with 4% paraformaldehyde for 20 min at room temperature. Immunostaining was performed using the same protocol as for brain sections (described below).

As for brain sections, sagittal cryosections were prepared from one brain hemisphere that had been flash-frozen in isopentane and stored at −80 °C. Sections (16 μm thick) were cut using a cryostat at −20 °C and fixed in 4% paraformaldehyde for 10 min at room temperature. After rinsing in PBS, tissue was permeabilized with 0.1% Triton X-100 in PBS (PBST) for 10 min, followed by blocking in 5% normal goat serum in PBST for 1 h at room temperature.

For single immunofluorescence staining, sections were incubated overnight at 4 °C with primary antibodies diluted 1:1000 in blocking buffer: anti-phospho-TDP-43 (Ser409/410) (Proteintech, 22309-1-AP), anti-GFAP (Proteintech, 16825-1-AP), and anti-6 × His-tag (Invitrogen, PA1-938-B). For co-immunofluorescence staining, sections were incubated overnight at 4 °C with a combination of anti-6 × His-tag (Proteintech, 66005-1-Ig, 1:500) and anti-TDP-43 C-terminal (Proteintech, 12892-1-AP, 1:1000) antibodies diluted in blocking buffer.

Following three washes in PBST, appropriate Alexa Fluor-conjugated secondary antibodies were applied for 1 h at room temperature in the dark, followed by three PBST washes. Nuclei were counterstained with Hoechst 33,342 for 10 min. Slides were rinsed with PBS, coverslipped with ProLong Diamond Antifade Mountant (Invitrogen), and stored protected from light.

Single immunofluorescence images were acquired using a slide scanner at 40 × magnification for pTDP-43, C-terminal TDP-43, and 6 × His-tag staining, and at 20 × magnification for GFAP staining. Image focus was set using the DAPI signal as reference. Co-immunofluorescence images were acquired on a SP8 confocal laser-scanning microscope (Leica Microsystems) using a 20 × objective and sequential acquisition mode to avoid spectral bleed-through; images were processed using Leica Application Suite X (LAS X) software.

Quantitative image analysis was performed using QuPath software. GFAP immunoreactivity was quantified as the percentage of stained area (% area) within predefined regions of interest corresponding to the cortex. Co-localization between TDP-43 and DAPI signals was assessed to determine the nuclear versus cytoplasmic distribution of TDP-43 and pTDP-43. Co-localization between 6 × His-tag and TDP-43 signals in co-immunofluorescence sections was evaluated by visual inspection of the merged channel, with overlapping signals in Tg-B1 sections compared to WT and Tg-13R4 negative controls.

### Proximity ligation assay on brain sections

*In situ* interaction between the 6 × His-tagged scFv B1 intrabody and TDP-43 was assessed by Proximity Ligation Assay (Duolink™ In Situ, Sigma-Aldrich) on brain sections from mice sacrificed 6 months post-injection. Brain sections were fixed in 4% paraformaldehyde (PFA) with 4% sucrose for 20 min at room temperature and permeabilized with 0,1% Triton X-100 in PBS for 10 min. Blocking was done according to manufacturer's instructions. Brain sections were incubated overnight at 4 °C with the two primary antibodies: anti-6 × His (Proteintech, 66005-1-Ig) and anti-TDP-43 C-terminal (Proteintech, 12892-1-AP). Species-specific PLA PLUS and MINUS probes were then applied, followed by ligation and amplification steps according to the manufacturer's instructions. A fluorescent signal is generated only when the two targeted epitopes lie within 40 nm of each other, reflecting a direct interaction or close molecular proximity between the two proteins. Slides were mounted with ProLong Diamond Antifade Mountant with DAPI (Invitrogen) and imaged by confocal microscopy (SP8, Leica) using sequential acquisition at 20 × magnification.

### RNA-seq

Total RNA was extracted from lyophilized mouse brain tissue and assessed for integrity using the Agilent TapeStation. Library preparation was performed using the Illumina Stranded Total RNA Prep with Ribo-Zero Plus according to the manufacturer's protocol. Library quality was verified using the Agilent TapeStation and concentrations were quantified using a Qubit fluorometer. Sequencing was carried out on an Illumina NextSeq 550 system, generating paired end (2 x 75 bp) reads with a depth of approximately 50 million reads per sample. This sequencing strategy was chosen to allow detection of alternative splicing events and cryptic exons.

Raw sequencing reads were first processed using FastQC (v0.12.1) and subsequently filtered with fastp (v0.24.0) [10] to remove adapter sequences and low-quality reads. FastQC and fastp reports were aggregated with MultiQC (v1.31) [11] to provide an integrated overview of sequencing quality across all samples. Potential contamination was assessed with FastQ Screen (v0.16.0) [12] against reference genomes including mouse, human, *Escherichia coli*, mycoplasma, and rRNA sequences. Reads were then aligned to the mouse reference genome GRCm39 using STAR (v2.7.11b) [13] with the two-pass mapping strategy and GENCODE gene annotation (vM38). Gene-level quantification was performed using STAR's quantMode GeneCounts option, which counts exonic reads based on the provided GTF annotation. Differential gene expression analysis was conducted in R (v4.3.3) using DESeq2 (v1.42.2) [14], with the design formula including the experimental group factor. Statistical significance was assessed using Wald test implemented in DESeq2, and p-values were adjusted for multiple testing using the Benjamini–Hochberg method. Volcano plots were generated in R using ggplot2 (v3.5.2) to visualize results, using the following thresholds: raw p-value <0.05 and |log2FoldChange| ≥ 0.585 (corresponding to a fold change ≥1.5).

### ^18^FDG- and ^18^F-DPA-714 PET imaging

All animals from the 2nd terminal cohort underwent a 4-day habituation phase prior to 18F-FDG imaging. During this period, each subject was exposed daily to a mock handling session reproducing the experimental procedures without radioactivity. This included simulated intraperitoneal (i.p.) injections and acclimation to the experimental environment to minimize stress during imaging. Animals were fasted overnight (<15h) before the imaging session.

18F-FDG (Curium Pharma, France) was administered intraperitoneally (i.p., 10.56 ± 0.48 MBq/mice) while the animals were awake. Following injection, the tracer was taken up by cells proportionally to their metabolic activity during a 45-min uptake period. After uptake, the mouse was anesthetized (isoflurane gas, 3% induction then 1.8–2.5% for maintenance), placed on a heating pad, and centered in the field of view of the IRIS PET/CT camera (Inviscan, France). A CT-scan and a list-mode PET acquisition of 30 min (6 frames of 5 min) starting 60 min after tracer injection were performed. Because 18F-FDG is an irreversible radiotracer, the resulting images primarily reflect tracer accumulation during the 45-min awake uptake period and are only minimally influenced by the anesthesia administered during scanning.

For the 18F-DPA-714 protocol, each subject was first anesthetized with isoflurane as described previously. A tail catheter was inserted using a 30G needle connected to PE-10 tubing. 18F-DPA-714 (CERRP, Tours) was administered intravenously (i.v., 8.59 ± 0.32 MBq/mouse) through the catheter, followed by a saline flush to ensure complete delivery of the tracer. Approximately 2 min later, the catheter was removed, anesthesia was stopped, and the mouse was allowed to recover in its cage for 25 min. Then, the mouse was re-anesthetized with isoflurane for PET acquisition. A CT scan and a 30-min PET scan were performed from 30 min post-injection to 60-min post-injection.

During the experiments, the respiratory rate and body temperature of each animal were monitored and kept as constant as possible (70 respirations per minute and 37 °C, respectively). List-mode scans were rebinned into 6 frames of 300 s, corrected for random and scatter, and images were reconstructed using a 3-D OSEM algorithm into voxels of 0.855 x 0.855 x 0.855 mm3. Data were corrected from partial volume effects using a Van Cittert reblurred approach for each time frame before summing over the entire acquisition. The registration of individual CT imaging into Mirrione template space was accomplished as a deformable transformation using PMOD v4.4 software (PMOD Technologies Ltd, Switzerland). The transformation learnt for each scan was applied to the PET imaging.

### Metabolomics analysis

The methodology used for this study was based on that proposed by our group for sample preparation, data preprocessing and processing [15]. Two modes of acquisition were used: C18 and hydrophilic interaction liquid chromatography (HILIC).

Brain and spinal cord samples were lyophilized for 48hrs. After which dry weight of the samples was measured, and 1000 μL of MeOH/dH_2_O (methanol/distilled water, 5:5) was added. The samples were vortexed for 5 s, shaken (30 min at 4 °C) and centrifuged (15,000 g, 15 min at 4 °C). Later, 900 μL of supernatant was collected and transferred into 2 tubes depending on the acquisition used. For the C18 mode: evaporation of 450 μL of the sample previously prepared (speed vac 4 h/40 °C), then 100 μL ACN/dH_2_O (acetonitrile/distilled water, 2:8) was added. Samples were centrifuged (15,000 g, 10 min/4 °C), and then 80 μL was transferred into a 96-well plate. Collection of 5 μL was done for the QC pool and sample injection of 5 μL was done for C18+ and C18- modes. For HILIC: evaporation of 450 μL of the sample previously prepared (SpeedVac 4hrs/40 °C), then addition of 100 μL ACN/dH_2_O (2:8), samples were transferred into amber vials. 5 μL was collected for the QC pool and 5 μL of sample injection was injected for the HILIC + mode.

For the dried blood spots (DBS): two 3 mm punches were extracted with 200 μL of ACN/MeOH/dH_2_O (4:4:2), then the sample was vortexed for 1 min. After 1 h planar agitation at high speed, the sample was centrifuged (10,000×*g* for 5 min/4 °C). Later, 150 μL of supernatant were aliquoted and dried under vacuum (SpeedVac) for 2 h/40 °C. The dried extract was dissolved in 75 μL of solvent (C18 ± = 100 μL ACN/dH_2_O (2:8); HILIC + = 100 μL ACN/dH2O (2:8). Samples were then vortexed for 1 min and left on planar agitation for 20 min at high speed. Ten microliters of each sample were injected into the mass spectrometer.

Mass spectrometry analysis was based on an UPLC Ultimate WPS-3000 system (Dionex, Germany) coupled to a Q-Exactive mass spectrometer (Thermo Fisher Scientific, Bremen, Germany). Positive (ESI+) and negative (ESI−) electrospray ionization modes were used for this analysis. Liquid chromatography used 2 different columns: a Phenomenex Kinetex 1.7 μm XB – C18 column (150 mm x 2.10 mm) maintained at 55 °C and HILIC column (150 mm x 2.10 mm, 100 Å), maintained at 40 °C. The mobile phases were water +0.1% formic acid/ACN +0.1% formic acid with a flow rate of 0.4 mL/min for C18; and water + formiate NH4 (10 mM) + 0.1% formic acid/ACN + formiate NH4 (10 mM) +0.1% formic with a flow rate of 0.4 mL/min for HILIC. The instrument operated at 70,000 resolutions during the full-scan acquisition, which ranged from 58 to 870 m/z.

The validation of the pre-analytical and analytical steps of the experiment was based on findings of QC samples (mix of samples analyzed). Coefficients of variation [CV% = (the standard deviation/mean) x 100], were calculated from all compounds. Metabolites with a CV > 30% in QCs were excluded from the final dataset. QCs were analyzed at the beginning of the run, every 10 samples and at the end of the run.

A library of standard compounds (Mass Spectroscopy Metabolite Library (MSML®) of standards, IROA Technologies™) was used to present a targeted analysis, including the identification step of metabolites based on the following criteria (1) retention time within ±20 s of the standard reference, (2) exact measured molecular mass within a range of 10 ppm around the known molecular mass of the reference compound, and (3) correspondence between isotopic ratios of the metabolite and the standard reference. Calculation of signal value was performed via Xcalibur® software (Thermo Fisher Scientific, San Jose, CA) after integration of the chromatographic peak area corresponding to the selected metabolites.

### Neurofilament and TDP-43 quantification in plasma

Plasma levels of NfL and TDP-43 were measured two days prior to treatment initiation (t0), 4 months post-treatment (t1), and 9 months post-treatment (t2) in the longitudinal cohort of WT, Tg-13R4, and Tg-B1 mice. These blood samples were collected prospectively as part of a dedicated longitudinal biomarker protocol. Mice were treated at 4 months of age, such that t1 corresponds to early but established neurodegeneration (8-month-old mice) and t2 to the severe disease endpoint (13-month-old mice).

Plasma neurofilament-light (NfL) and TDP-43 were quantified on the Quanterix Simoa SR-X analyzer using the Simoa N4PD Advantage PLUS reagent kit (for NfL within the N4PD panel) and the Simoa TDP-43 kit according to the manufacturers’ instructions. Prior to analysis, frozen EDTA-plasma samples were thawed on ice, gently inverted to mix, and clarified by brief centrifugation to remove particulate matter; plasma was then processed and analyzed in technical duplicate for each analyte. Samples were assayed using the kit-provided calibrator concentrates, diluents, bead and detector reagents and run-specific controls (all supplied with each kit) and plates/programs were prepared on the SR-X following the kit instruction recommendations. Calibrators and QC controls were included on every run to permit curve fitting and run acceptance. Resulting concentrations were calculated by the SR-X software from the supplied calibrators, and the mean of duplicate wells was reported for each sample.

### Statistical analysis

The data for western blot, phenotypic changes, immunofluorescence, NfL, and TDP-43 were analyzed with the GraphPad Prism 10 software. Data were analyzed by 2-way ANOVA with Sidak's post hoc test for multiple comparisons, unpaired t-tests, or Mann-Whitney U tests. We performed Kolmogorov-Smirnov tests on quantitative datasets. If normality was not met, we adjusted our statistical approach to non-parametric methods. Results were considered significant when p-values<0.05.

For metabolomics analysis of the brain, univariate analysis was done using MetaboAnalyst 6.0 computational software to compare metabolite levels between the different groups. Differentially detected metabolites were identified using volcano plot analysis with False Discovery Rate (FDR) correction. As few metabolites were considered significant, we extended our study with a fold-change threshold of 1.3 and a p-value <0.5 to identify more metabolites. Multivariate analysis of metabolomic profiles was also conducted using MetaboAnalyst 6.0. Models were generated to compare: (i) Tg mice treated with scFv 13R4 vs WT mice (to evaluate the effect of a control AAV-mediated scFv), and (ii) Tg mice treated with scFv B1 versus Tg mice treated with scFv 13R4 (to evaluate the effect of our therapeutic candidate). Prior to analysis, data were log10-transformed and auto-scaled (mean-centered and divided by the standard deviation of each variable). Principal component analysis (PCA) was first performed to assess data quality, detect potential outliers, and evaluate metabolic variability among WT and treated Tg mouse groups. Orthogonal partial least-squares discriminant analysis (OPLS-DA) was used to evaluate group discrimination and to identify metabolites contributing most strongly to group separation. Pairwise OPLS-DA comparisons included Tg 13R4 vs WT, and Tg 13R4 vs Tg B1. Metabolites were ranked according to their Variable Importance in Projection (VIP) scores derived from the OPLS-DA models. Metabolites with VIP values greater than 1 were considered discriminant, and the top 20 discriminant metabolites were selected for further analysis. In line with the objectives of this study, common and distinct metabolites were compared across treated Tg-B1 and WT/Tg-13R4 groups in both brain and spinal cord tissues. Venn diagrams were generated using Venny 2.0.2 to visualize overlaps among significant metabolites identified through both univariate and multivariate analyses. After conducting metabolomics analyses and drawing a Venn diagram to compare all the groups, all the metabolites common to the Tg-13R4 vs. WT and Tg-13R4 vs. Tg-B1 comparisons were identified and consequently considered relevant in reflecting the ability of our candidate B1 to counteract the effects of TDP-43 pathology. Finally, pathway enrichment and metabolic pathway analyses were performed using MetaboAnalyst 6.0 based on the KEGG pathway database (http://www.genome.jp/kegg/pathway.html). These analyses were conducted using the top 20 discriminant metabolites identified across all OPLS-DA models and univariate statistical analysis.

For all the longitudinal parameters in the longitudinal cohort of mice (clinical factors and DBS metabolomics profiles), the results were analyzed as a percentage of modification between two specific time points to provide a clear overview of the disease trajectory. Indeed, given the marked inter-individual variability among Tg mice, longitudinal analyses were performed using the evolution of quantitative parameters over time for each individual mouse. For body weight, grip strength, and rotarod performance, data were expressed as the percentage change relative to baseline values at t0. Parameters assessed using arbitrary scoring systems, including wire maneuver performance, tail position, and gait impairment, were analyzed using absolute scores rather than percentage change. The same software as previously mentioned were used.

For micro-PET data, a voxel-based analysis was used to assess the differences in cerebral 18F-FDG and 18F-DPA-714 uptakes between the averaged brains of Tg-13R4 vs WT and Tg-B1 vs Tg-13R4 mice. The regions of interest were derived from Mirrione's templates using PMOD v3.2 software (PMOD Technologies Ltd, Switzerland) and applied to Z-score and effect size maps to obtain the Z-score and *d* values in these areas (Accrombessi et al., 2022; Sawilowsky, 2009). Statistical analyses focused on inter-group comparisons using a two-tail unpaired Student t-test. Differences were considered significant when p < 0.01 for signals of at least 50 contiguous voxels, with *d* values of at least 0.80.

## Results

### B1 effect on NF-kB in NSC-34 cells

Among the tested scFvs, only B1, D7 and 13R4 showed robust expression in cells, as confirmed by western blot (Supplementary Fig. 1a). For this reason, only these three scFvs were included in subsequent *in vitro* analyses.

Immunofluorescence analysis revealed good expression of these scFvs and TDP-43 in NSC-34 cells (Supplementary Fig. 1b). The scFvs were predominantely localized in the cytoplasm. Despite the clear cytoplasmic signal of TDP-43 observed by immunofluorescence, western blot analysis showed no significant change in total TDP-43 levels in NSC-34 cells (Supplementary Fig. 2). Additionally, TDP-43 overexpression resulted in the appearance of the 35 kDa C-terminal fragment of TDP-43. The scFvs did not significantly affect the overall levels of TDP-43.

Interestingly, the overexpression of TDP-43 induced a 2.68 ± 0.9-fold increase in p65 levels compared to the mock (empty plasmid) condition. Co-expression of the scFv B1 significantly reduced this TDP-43-induced increase with restoring p65 levels close to the mock condition (1.26 ± 0.27, ∗*p* = 0.03). This effect was not observed for the other scFvs (Fig. 2). Based on these results, the scFv B1 and the control scFv 13R4 were selected for further evaluation in an ALS mouse model.

**Fig. 2:**
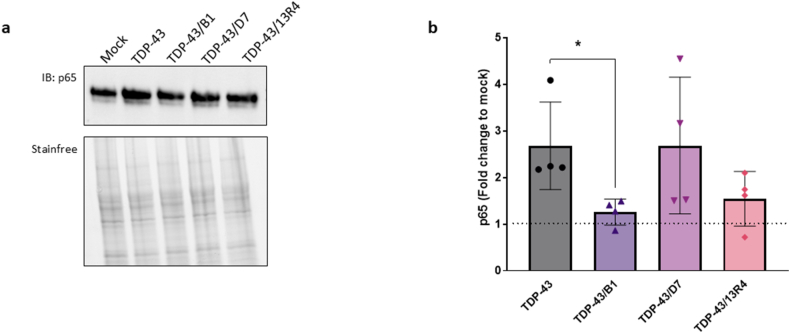
**TDP-43 activates NF-κB and B1 reverses it (a).** Immunoblot of p65 in NSC34 cells after 48 h of overexpression. Mock: transfection of empty vector. TDP-43: transfection of TDP-43 and empty vector. TDP/B1, TDP/D7, TDP/Control: co-transfection of TDP-43 and corresponding intrabody. **b)** Quantification of the active subunit of NF-κB, p65, in total lysate normalized to stain-free lanes. Values were normalized to the mock condition (dotted line). ∗*p* = 0.036, Mann-Whitney test; N = 4.

### Phenotypic characterization and longitudinal follow-up

Two days before treatment initiation (t0), Tg mice were already symptomatic. Compared with WT mice, both Tg mice treated with the control scFv (Tg-13R4) and Tg mice treated with scFv B1 (Tg-B1) displayed significant impairments across all assessed phenotypic parameters, including reduced body weight, decreased grip strength, shorter latency to fall in the rotarod test, impaired performance in the wire maneuver, abnormal tail position, and increased gait impairment scores (Fig. 3).

**Fig. 3:**
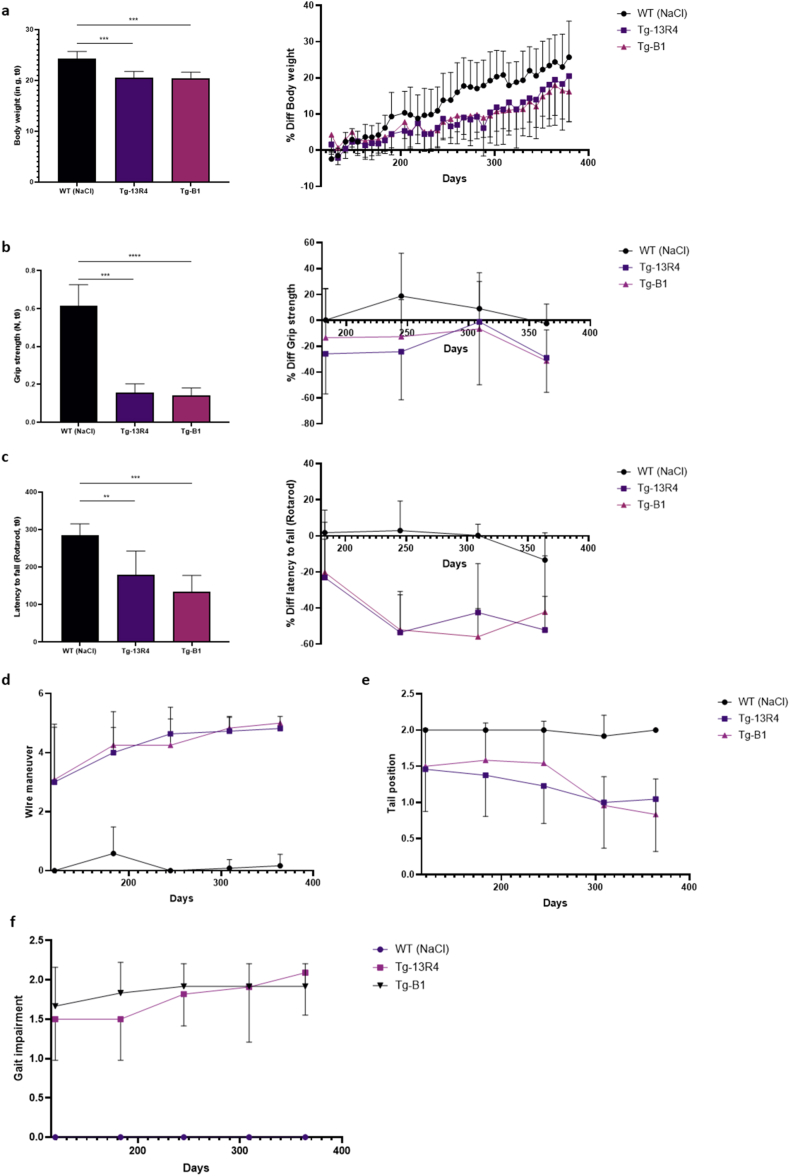
**Longitudinal phenotypic follow-up from before treatment to 9 months post-treatment in Tg and WT mice. a)** Body weight at baseline (T0) (left panel) and percentage change in body weight relative to T0 over the 9-month follow-up (right panel), **b)** Grip strength at baseline (T0) (left panel) and percentage change in grip strength relative to T0 over time (right panel), **c)** Rotarod performance at baseline (T0) (left panel) and percentage change in rotarod performance relative to T0 (right panel), **d)** Wire maneuver, **e)** Tail position, and **f)** gait impairment scores evaluated over time using an arbitrary scale.

Longitudinal follow-up revealed no treatment-related differences between Tg-B1 and Tg-13R4 mice for body weight, grip strength, or rotarod performance (Fig. 3a). Similarly, no treatment-related differences were observed for grip strength or rotarod performance, with both Tg groups showing comparable progression profiles throughout the study period (Fig. .3b–c).

As expected, Tg mice differed markedly from WT mice for all wire maneuver performances, tail position and gait impairment. However, no significant differences were observed between Tg-B1 and Tg-13R4 mice at any time point (Fig. 3d–f).

### Longitudinal effect of the scFv B1 on NfL and TDP-43 in the plasma

At t0, both transgenic groups exhibited significantly elevated plasma NfL levels compared with WT mice, indicating the presence of ongoing neurodegeneration at the time of treatment initiation. Longitudinal analysis revealed that NfL levels remained relatively stable over time in all groups, with no significant changes observed between t0, t1, and t2, and no differences were detected between Tg-13R4 and Tg-B1 mice at any time point (Fig. 4a).

**Fig. 4:**
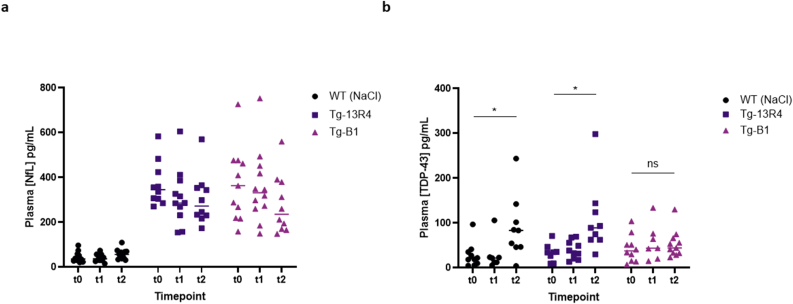
**Plasma biomarker measurements (NfL and TDP-43) quantified by Simoa at 3 longitudinal timepoints.** Absolute plasma TDP-43 concentrations (pg/mL) measured at t0, t1, and t2 for the three groups. Plasma samples were collected at t0 (2 days prior to injection), t1 (4 months post-injection), and t2 (endpoint, 9 months post-injection). Results are represented as mean ± SD. Mixed-Effects analysis (N = 10–11 per group).

Plasma TDP-43 levels showed a distinct profile. At t0 and t1, TDP-43 concentrations were comparable across all groups and did not differ significantly from WT levels. For instance, at t0, plasma TDP-43 levels were 27.94 ± 26.99 pg/mL in WT mice, 33.36 ± 20.02 in Tg-13R4 mice, and 42.56 ± 30.41 in Tg-B1 mice. In contrast, at t2, a significant increase in plasma TDP-43 was observed in WT and Tg-13R4 mice, whereas Tg-B1 mice did not show such an increase. At t2, plasma TDP-43 levels reached 89.56 ± 69.83 pg/mL in WT mice and 108.6 ± 78.73 in Tg-13R4 mice, compared with 52.97 ± 30.2 in Tg-B1 mice (Fig. 4b).

Together, these results indicate that while scFv B1 treatment had no effect on plasma NfL levels but was associated with a prevention of the rise in plasma TDP-43 levels at later time points compared with control-treated Tg mice. Individual data points are overlaid on all NfL and TDP-43 bar graphs (Fig. 4) to allow direct assessment of inter-individual dispersion.

### Longitudinal metabolomic analyses

In an independent terminal cohort analyzed 9 months post-treatment, metabolomic analyses were performed on dried blood spots (DBS) collected before injection (T0), 4 months post-injection (T1) and prior to sacrifice (T2), comparing WT, Tg-13R4, and Tg-B1 mice. DBS metabolomics enabled the detection of 235 metabolites across samples. To assess metabolomic changes associated with disease progression, we first compared Tg mice treated with the control scFv 13R4 to age-matched WT animals at T1, T2 and T3. We then compared the differential metabolites between T1 and T2, T1 and T3, T2 and T3 and calculated a percentage of change between each time for each metabolite. Univariate analysis was performed but did not identify statistically significant metabolites after multiple-testing correction.

Multivariate analysis was therefore performed to identify the top 20 metabolites contributing most strongly to group discrimination (e.g. Tg13R4 vs WT and Tg-13R4 vs Tg-B1 at T1 vs T2, T1 vs T3 and T2 vs T3) based on VIP scores. Among these top 20 metabolites at T1 vs T2, five were common to both comparisons (Tg-13R4 vs WT and Tg-13R4 vs Tg-B1). N-Formylglycine and Taurocholic acid were increased in Tg-13R4 mice compared to Tg-B1 mice, indicating that Tg-B1 mice exhibited levels closer to WT. Moreover, Spermidine and Thymine were decreased in Tg-13R4 mice relative to WT and showed the same direction change when comparing Tg-13R4 mice compared to Tg-B1 mice, again reflecting a shift toward WT levels in Tg-B1 animals. However, 2-Quinolinecarboxylic acid was increased in Tg-13R4 mice compared to WT and decreased in Tg-13R4 mice compared to Tg-B1 mice (Fig. 5).

**Fig. 5:**
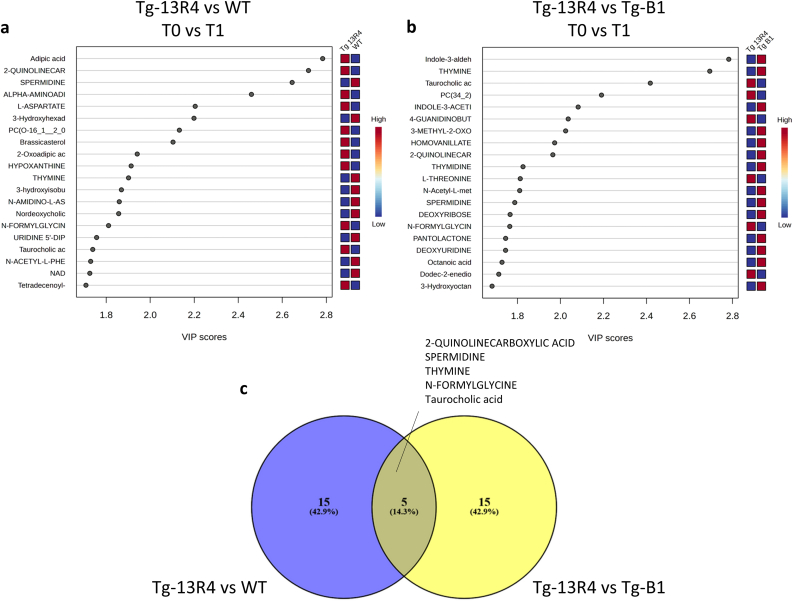
**Longitudinal multivariate metabolomic profiling in mouse brains before AAV administration and 4 months after the injection: a-b)** Top 20 metabolites ranked by Variable Importance in Projection (VIP) scores from oPLS-DA models comparing Tg-13R4 vs WT mice (A) and Tg-13R4 vs Tg-B1 mice (B) differential metabolites between before the injection (T0) and 4 months after the injection (T1). Higher VIP values indicate a stronger contribution of each metabolite to the discrimination between the studied groups. **c)** Venn diagram illustrating the overlap and specificity of the top 20 VIP metabolites identified by oPLS-DA in the Tg-13R4 vs WT and Tg-13R4 vs Tg-B1 differential metabolites between before the injection (T0) and 4 months after the injection (T1) comparisons. Shared metabolites correspond to those altered in both comparisons, whereas non-overlapping sections indicate metabolites unique to each comparison.

To continue, at T1 vs T3, seven metabolites were common to both comparisons. Indole-3-propionic acid, Alpha-aminoadipate, Indoleacrylic acid and Deoxyribose were increased in Tg-13R4 mice compared to WT and Tg-B1 mice, indicating that Tg-B1 mice exhibited levels closer to WT. On the contrary, Lumichrome, D-Panthotenic acid and L-Tryptophan were decreased in Tg-13R4 mice compared to Tg-B1 mice, indicating a normalization toward WT levels in Tg-B1 mice (Fig. 6).

**Fig. 6:**
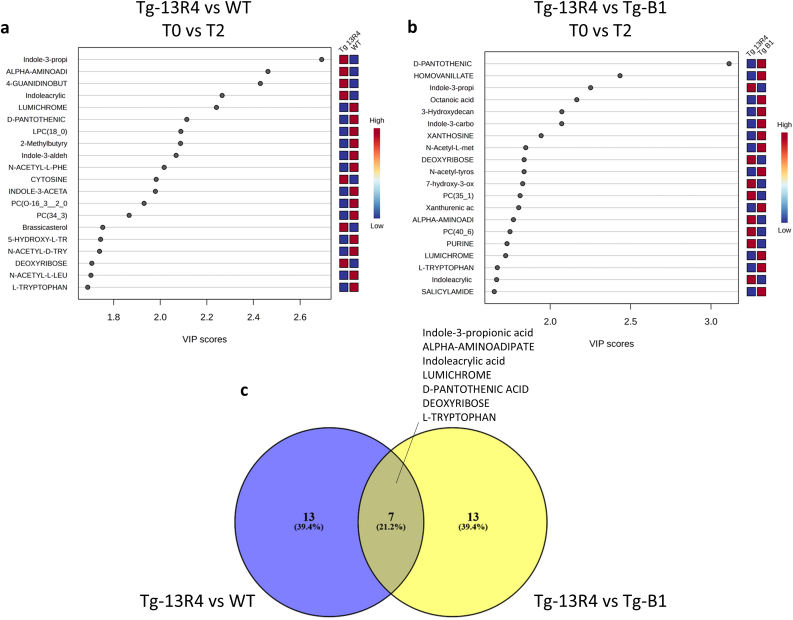
**Longitudinal multivariate metabolomic profiling in mouse brains before AAV administration and before sacrifice: a-b)** Top 20 metabolites ranked by Variable Importance in Projection (VIP) scores from oPLS-DA models comparing Tg-13R4 vs WT mice (A) and Tg-13R4 vs Tg-B1 mice (B) differential metabolites between before the injection (T0) and before sacrifice (T2). Higher VIP values indicate a stronger contribution of each metabolite to the discrimination between the studied groups. **c)** Venn diagram illustrating the overlap and specificity of the top 20 VIP metabolites identified by oPLS-DA in the Tg-13R4 vs WT and Tg-13R4 vs Tg-B1 differential metabolites between before the injection (T0) and before sacrifice (T2) comparisons. Shared metabolites correspond to those altered in both comparisons, whereas non-overlapping sections indicate metabolites unique to each comparison.

Moreover, at T2 vs T3, six metabolites were common to both comparisons. Indoleacrylic acid was again increased in Tg-13R4 mice compared to WT and to Tg-B1 mice. To continue, 2-Methylbutyrylglycine, L-Methionine, Lactate, L-Alanine, L-Glutamine and Creatine were decreased in Tg-13R4 mice compared to WT and showed the same direction of change in Tg-13R4 mice compared to Tg-B1 mice, indicating a normalization again toward WT levels in Tg-B1 animals (Fig. 7).

**Fig. 7:**
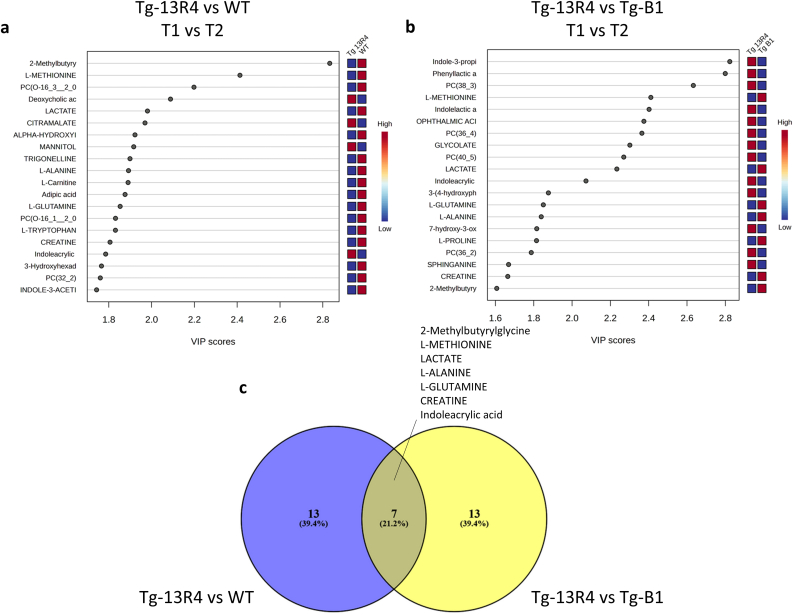
**Longitudinal multivariate metabolomic profiling in mouse brains 4 months after AAV administration and before sacrifice: a-b)** Top 20 metabolites ranked by Variable Importance in Projection (VIP) scores from oPLS-DA models comparing Tg-13R4 vs WT mice (A) and Tg-13R4 vs Tg-B1 mice (B) differential metabolites between 4 months after injection (T1) and before sacrifice (T2). Higher VIP values indicate a stronger contribution of each metabolite to the discrimination between the studied groups. **c)** Venn diagram illustrating the overlap and specificity of the top 20 VIP metabolites identified by oPLS-DA in the Tg-13R4 vs WT and Tg-13R4 vs Tg-B1 differential metabolites between 4 months months after injection (T1) and before sacrifice (T2) comparisons. Shared metabolites correspond to those altered in both comparisons, whereas non-overlapping sections indicate metabolites unique to each comparison.

### Terminal cohort exploration at 6 months post treatment

#### Viral DNA quantification in tissues

Droplet digital PCR (ddPCR) analysis was performed on genomic DNA extracted from lyophilized brain, spinal cord and liver tissues to quantify AAV genome copies. As expected, no AAV copies were detected in the WT group, confirming the absence of vector contamination or background signal in untreated samples. In contrast, brains from AAV-treated mice analyzed six months post-injection showed a robust and consistent presence of vector genomes (Fig. 8). Quantification revealed an average of approximately one AAV copy per diploid genome, with values of 1.2 ± 0.1 for the Tg-13R4 group and 1.699 ± 0.40 for the Tg-B1 group. A similar distribution was observed in the spinal cord, where AAV genome copies averaged 0.9 ± 0.1 per diploid genome in Tg-13R4 mice and 1.0 ± 0.16 in Tg-B1 mice. No AAV genomes were detected in the liver, with 0.08 ± 0.06 for the Tg-13R4 group and 0.08 ± 0.05 for the Tg-B1 group, indicating a lack of peripheral vector dissemination.

**Fig. 8:**
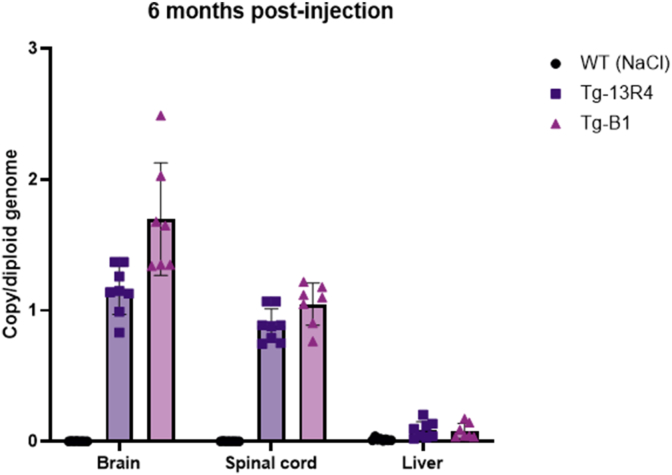
**Quantification of AAV viral DNA copies in the brain, spinal cord and liver at 6 months post-treatment.** Viral vector genome levels are expressed as viral DNA copies per diploid genome. Each point represents an individual animal, and bars represent mean ± SD. Mann-Whitney test, N = 7–8.

#### Metabolomics profile

In an independent terminal cohort analyzed at six months post-treatment, metabolomic analyses were performed in the brain and spinal cord, comparing WT, Tg-13R4, and Tg-B1 mice. Metabolomic analyses of the brain tissue at six months post-treatment allowed us to detect 266 metabolites across the samples. To identify metabolic changes associated with disease progression, we first compared Tg mice treated with the control scFv 13R4 to age-matched WT animals. Univariate analysis identified several metabolites of interest, although none remained significant after adjusting for multiple testing (Fig. .9a–b).

**Fig. 9:**
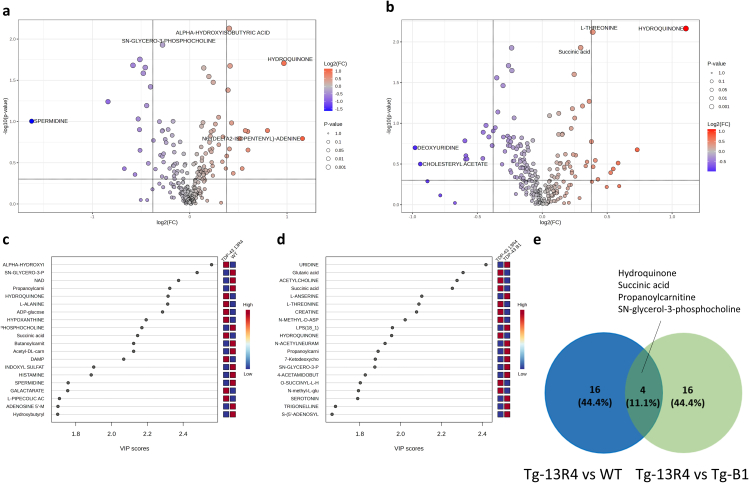
**Univariate and multivariate metabolomic profiling in mouse brains after 6 months of AAV administration: a-b)** Volcano plot showing differential metabolite abundance between brains of Tg-13R4 vs WT mice (A) and Tg-13R4 vs Tg-B1 mice (B) after 6 months of treatment. The log_2_ fold change of relative metabolite intensities is plotted against the −log_10_ of the p-value adjusted for multiple testing using the false discovery rate (FDR). Each point represents an identified metabolite. Significantly increased metabolites (log_2_FC > 1.3, FDR <0.5) are shown in red, significantly decreased metabolites (log_2_FC < −1.3, FDR <0.5) in blue, while non-significant metabolites are shown in grey. **c-d)** Top 20 metabolites ranked by Variable Importance in Projection (VIP) scores from oPLS-DA models comparing Tg-13R4 vs WT mice (C) and Tg-13R4 vs Tg-B1 mice (D) after 6 months of treatment. Higher VIP values indicate a stronger contribution of each metabolite to the discrimination between the studied groups. **e)** Venn diagram illustrating the overlap and specificity of the top 20 VIP metabolites identified by oPLS-DA in the Tg-13R4 vs WT and Tg-13R4 vs Tg-B1 comparisons. Shared metabolites correspond to those altered in both comparisons, whereas non-overlapping sections indicate metabolites unique to each comparison.

In the brain, multivariate analysis was therefore used to identify the top 20 metabolites contributing most strongly to group discrimination (e g Tg-13R4 vs WT and Tg-13R4 vs Tg-B1) based on VIP scores. Among these top 20 metabolites, five were common to both comparisons (Tg-13R4 vs WT and Tg-13R4 vs Tg-B1). Hydroquinone and succinic acid were increased in Tg-13R4 mice compared to WT animals and showed a similar increase in Tg-13R4 mice when compared to Tg-B1 mice, indicating that Tg-B1 mice exhibited levels closer to WT. Conversely, Propanoylcarnitine and SN-Glycerol-3-Phosphocholine were decreased in Tg-13R4 mice compared to WT and showed the same direction of change in Tg-13R4 mice compared to Tg-B1 mice, again indicating a normalization toward WT levels in Tg-B1 animals (Fig. 9c–e).

In the spinal cord, the same multivariate approach identified the top 20 VIP metabolites for each comparison. Only two metabolites were shared between the Tg-13R4 vs WT and Tg-13R4 vs Tg-B1 comparisons. Hydroxyadipic acid was decreased in Tg-13R4 mice compared to WT and increased in Tg-13R4 mice compared to Tg-B1, indicating opposite directions of change in the two comparisons. Phosphocholine was increased in Tg-13R4 mice compared to WT and showed a similar increase in Tg-13R4 mice compared to Tg-B1, with Tg-B1 mice displaying levels closer to WT (Fig. 10).

**Fig. 10:**
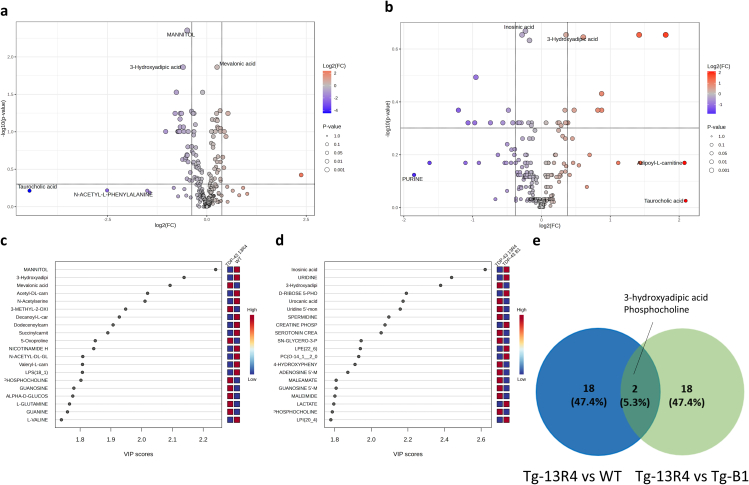
**Univariate and multivariate metabolomic profiling in mouse spinal cords after 6 months of AAV administration: a-b)** Volcano plot showing differential metabolite abundance between spinal cords of Tg-13R4 vs WT mice (A) and Tg-13R4 vs Tg-B1 mice (B) after 6 months of treatment. The log_2_ fold change of relative metabolite intensities is plotted against the −log_10_ of the p-value adjusted for multiple testing using the false discovery rate (FDR). Each point represents an identified metabolite. Significantly increased metabolites (log_2_FC > 1.3, FDR <0.5) are shown in red, significantly decreased metabolites (log_2_FC < −1.3, FDR <0.5) in blue, while non-significant metabolites are shown in grey. **c-d)** Top 20 metabolites ranked by Variable Importance in Projection (VIP) scores from oPLS-DA models comparing Tg-13R4 vs WT mice (C) and Tg-13R4 vs Tg-B1 mice (D) after 6 months of treatment. Higher VIP values indicate a stronger contribution of each metabolite to the discrimination between the studied groups. **e)** Venn diagram illustrating the overlap and specificity of the top 20 VIP metabolites identified by oPLS-DA in the Tg-13R4 vs WT and Tg-13R4 vs Tg-B1 comparisons. Shared metabolites correspond to those altered in both comparisons, whereas non-overlapping sections indicate metabolites unique to each comparison.

#### Immunofluorescence

Immunofluorescence analysis was assessed on the terminal cohort mice brains (six months). They confirmed strong CNS expression of the AAV-delivered scFv B1. The scFv signal appeared as a diffuse cytoplasmic staining pattern, with higher intensity in subcortical regions and in areas adjacent to the ventricles, consistent with widespread vector distribution (Fig. 11).

**Fig. 11:**
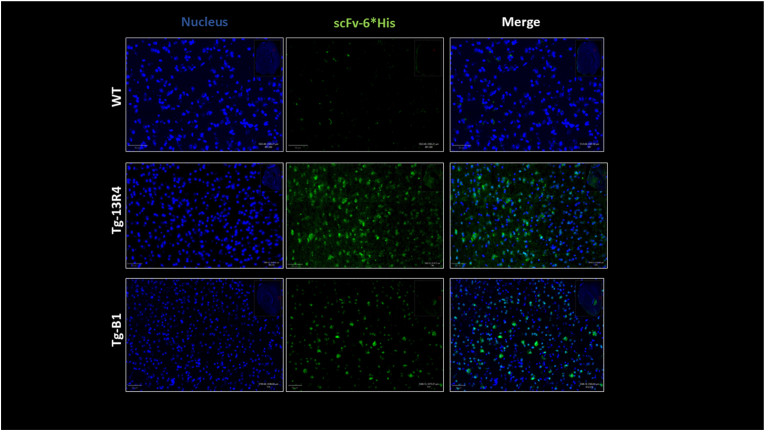
**Immunofluorescence detection of 6 × His-tagged scFv in mouse brain.** Representative immunofluorescence images showing 6 × His-tagged scFv signal in sagittal brain sections from WT, Tg-13R4, and Tg-B1 mice. Nuclei are counterstained with DAPI (blue), and 6 × His-scFv is shown in green. (scale bar = 50 μm).

In Tg-B1 brain sections, the 6 × His-tagged scFv signal co-localised with TDP-43, as evidenced by overlapping fluorescence in the merged channel. As expected, no 6 × His signal was detected in WT sections, and although 6 × His signal was present in Tg-13R4 sections confirming successful AAV transduction, no co-localization with TDP-43 was observed, consistent with the lack of TDP-43 binding specificity of the control intrabody. Together, these results provide *in situ* evidence that scFv B1 reaches its target in the CNS following AAV-mediated delivery (Fig. 12).

**Fig. 12:**
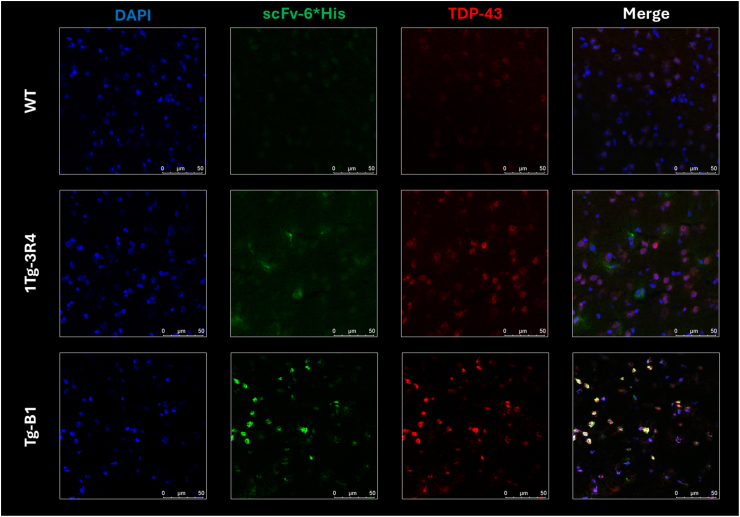
**Intrabody B1 co-localizes with TDP-43 in the brains of ALS transgenic mice**. Immunofluorescence staining of brain sections from WT, Tg-13R4, and Tg-B1, sacrificed 6 months post-treatment. Nuclei are counterstained with DAPI (blue), TDP-43 is shown in red and 6xHis signal is shown in green. White arrows indicate TDP-43. Red arrows indicate co-localization of scFv B1 with TDP-43-positive foci. Scale bar = 50 μm.

Astroglial reactivity, assessed by GFAP staining, did not differ between WT, Tg-13R4, and Tg-B1 groups, suggesting that neither disease progression at 10 months of age nor scFv B1 and scFv 13R4 expression markedly altered baseline astrocytic activation (Fig. 13).

**Fig. 13:**
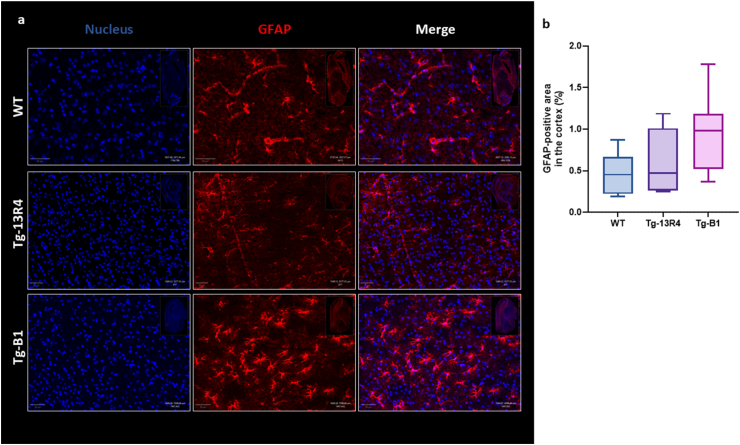
**Immunofluorescence detection of GFAP in mouse brain. a)** Representative immunofluorescence images showing GFAP signal in sagittal brain sections from WT, Tg-13R4, and Tg-B1 mice. Nuclei are counterstained with DAPI (blue), and GFAP is shown in red (scale bar = 50 μm). **b) Quantification of GFAP-positive area in the cortex.** Box plot showing the percentage of GFAP-positive area in cortical sections from WT, Tg-13R4, and Tg-B1 mice. Each box represents the interquartile range, the horizontal line indicates the median, and whiskers show minimum to maximum values.

Quantification of phosphorylated TDP-43 (pTDP-43)–positive cells revealed no reduction in overall accumulation following B1 treatment. Tg-13R4 mice displayed 76 ± 11.71 pTDP-43^+^ cells, whereas Tg-B1 mice showed 88.53 ± 4.92 pTDP-43^+^ cells (p = 0.0012), indicating a modest but statistically significant increase rather than a decrease in detectable pTDP-43 signal (Fig. 14a–b).

**Fig. 14:**
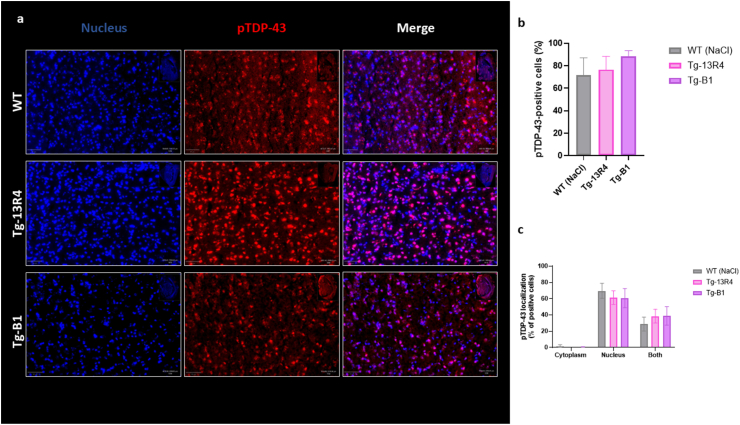
**Immunofluorescence detection of pTDP-43 in mouse brain. a)** Representative immunofluorescence images showing pTDP-43 signal in sagittal brain sections from WT, Tg-13R4, and Tg-B1 mice. Nuclei are counterstained with DAPI (blue), and pTDP-43 is shown in red. (scale bar = 50 μm). **b) Quantification of pTDP-43-positive cells in the cortex.** Percentage of pTDP-43-positive cells in cortical sections from WT, Tg-13R4, and Tg-B1 mice. Results are represented as mean ± SD. **c) Localization of pTDP-43-positive cells in the cortex.** Percentage of pTDP-43-positive cells localization (cytoplasm, nucleus, both) in cortical section from WT, Tg-13R4 and Tg-B1 mice. Results are represented as mean ± SD.

Analysis of subcellular localization further demonstrated that B1 treatment did not alter the intracellular distribution of pTDP-43. Across groups, approximately 60% of pTDP-43 signal remained confined to the nucleus, while ∼40% was present in both the nucleus and cytoplasm. Fewer than 1% of cells exhibited exclusively cytoplasmic pTDP-43 (Fig. 14c). These findings indicate that despite robust expression, scFv B1 did not modify the overall pattern of pTDP-43 mislocalization *in vivo*.

#### Proximity ligation assay

To further investigate, at the molecular scale, the potential *in vivo* interaction between intrabody B1 and TDP-43 suggested by our co-immunofluorescence findings, we performed PLA on brain sections from mice sacrificed 6 months post-treatment. Using antibodies directed against the 6 × His tag (scFv) and TDP-43, a PLA signal was detected exclusively in brain sections from Tg-B1-injected mice, consistent with close molecular proximity between intrabody B1 and TDP-43 *in vivo* (Supplementary Fig. 3). All control conditions yielded negligible signal: no puncta were observed in WT mice, which lack any scFv transgene. Single-probe controls (anti-TDP-43 antibody only) and sections from Tg-13R4-injected mice showed only minimal residual signal, likely reflecting background autofluorescence from lipofuscin accumulation in aged tissue or the high endogenous TDP-43 expression characteristic of the transgenic model. Taken together, these results provide indirect, suggestive evidence of proximity between scFv B1 and TDP-43 at the molecular scale, in line with the IF findings, and are consistent with scFv B1 associating with mislocalized TDP-43 aggregates *in situ*.

#### RNA-sequencing analyses

RNA-sequencing was performed on brain tissue from this terminal second cohort to characterize transcriptional changes across experimental groups. In the comparison between Tg-13R4 and WT mice (Fig. 15), the most prominent changes consisted of increased expression of immune-related and immunoglobulin-associated transcripts. These included Igkc, Ighg1, Ighg2b, Ighg2c, Ighv3-6, Igkv17*-127,* and Jchain, as well as the complement component C4b and Nlrc5. Conversely, Tg-13R4 mice showed reduced expression of multiple predicted transcripts including Uox and Pappa2, relative to WT controls.

**Fig. 15:**
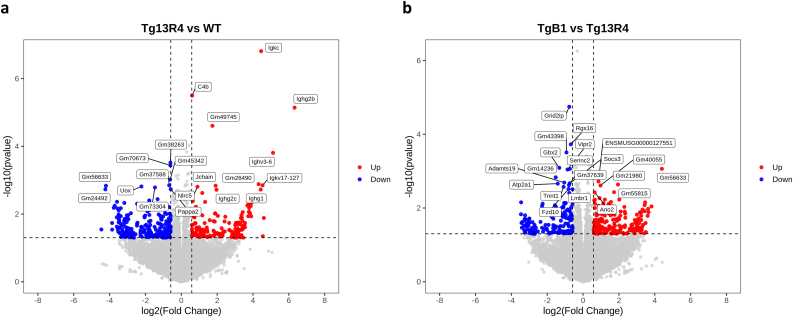
**Differential gene expression analysis in brain samples at 6 months post-treatment.** Volcano plots illustrating gene expression in brain samples comparing Tg-13R4 vs WT mice (A) and Tg-B1 vs Tg-13R4 mice (B). Each point represents an individual transcript. Thresholds used to define differential expression were a raw p-value <0.05 and an absolute log_2_ fold change ≥0.585, corresponding to a fold change ≥1.5. Transcripts meeting these criteria are highlighted, while non-significant transcripts are shown in grey.

Comparison of Tg-B1 versus Tg-13R4 mice revealed a distinct transcriptional profile. Among the transcripts upregulated in Tg-B1 mice were Ano2 and Socs3. Notably, a predicted gene, Gm56633, which was decreased in Tg-13R4 mice relative to WT, was increased in Tg-B1 mice compared to Tg-13R4, indicating an opposite direction of change between the two comparisons. In contrast, a larger number of transcripts were downregulated in Tg-B1 mice compared to Tg-13R4, including genes associated with neuronal signalling, development, and cellular function (Grid2ip, Gbx2, Adamts19, Atp2a1, Tnnt1, Lmbr1, Fzd10, Rgs16, Vipr2, Serinc2).

#### Effect of B1 on cerebral metabolism and neuroinflammation

On the same terminal cohort, ^18^FDG - and ^18^F -DPA-PET imaging were performed to assess cerebral glucose metabolism and TSPO density (translocator protein targeted by DPA-714) signals in WT, Tg-13R4, and Tg-B1 mice.

FDG-PET analysis revealed that Tg-13R4 mice exhibited significantly higher glucose metabolism in the prelimbic/infralimbic cortex, orbitofrontal cortex, striatum, periaqueductal grey, hippocampus, and mesencephalon compared to WT mice (z-score ranging from 3.33±0.20 to 3.74±0.58 corresponding to d-values ranging from 1.31 to 1.38 and p-values ranging from 0.0037 to 0.0006 in the PrL/IL and periacqueductal grey, respectively), while a lower signal was detected in the cerebellum (z-score = −4.05±0.80, d-value = 1.43, p = 0.0003) (Supplementary Fig. 4a). Treatment with the scFv B1 did not lead to a normalization of the metabolic alterations observed in Tg-13R4 at 6 months post-treatment, as no significant differences were detected in the regions showing altered metabolism in Tg-13R4 mice relative to WT. TG mice treated with the scFv B1 showed additional higher 18FDG uptake in the insular cortex (z-score = 3.45±0.27, d-value = 1.32, p = 0.0006) and lower 18FDG uptake values in the hippocampus (z-score = −3.45±0.24, d-value = 1.34, p = 0.0093) when compared to Tg-13R4 mice. Thus, B1 only showed a normalizing effect in metabolic activity in the hippocampus (Supplementary Fig. 4b).

Analyses of 18F-DPA-714 uptake showed significantly lower uptake in motor cortex, striatum, hypothalamus, and visual cortex of Tg-13R4 mice compared to WT animals (z-score comprised between −3.33±0.28 and −3.45±0.39 corresponding to d-values ranging from 1.28 to 1.31 and p-values ranging from 0.0019 to 0.0030 in the motor cortex and striatum, respectively. In contrast, a higher signal was detected in the insular cortex (z-score = 3.18±0.15, d-value = 1.25, p = 0.0070: Supplementary Fig. 4c). No significant differences in 18F-DPA-PET signal were detected when comparing Tg-B1 mice to Tg-13R4 mice, indicating that scFv B1 treatment did not significantly alter the microglial activity at 6 months post-treatment under the conditions tested (Supplementary Fig. 4d).

## Discussion

This study investigated the therapeutic potential of the anti-TDP-43 scFv B1. Using a combination of cellular assays and multi-modal analysis in both cellular and animal model of ALS, we aimed to determine whether B1 could mitigate TDP-43-associated proteinopathy. Across our experimental approaches, from cell culture to plasma biomarkers, PET imaging, metabolomics, and RNA-seq, our results reveal the consequences of TDP-43 dysregulation and the capacity of scFv B1 to modulate some aspects of this pathology.

### TDP-43 dysregulation activates inflammatory pathways which are partially attenuated by B1

In NSC-34 cells, TDP-43 overexpression promoted its cytoplasmic mislocalization and the formation of a 35-kDa C-terminal fragment, reflecting early pathological processing rather than an increase in total TDP-43 levels, likely due to autoregulatory mechanisms [16,17]. This dysregulation resulted in a marked increase in p65 levels. Previous studies have shown that TDP-43 dysregulation lead to an increase in NF-kB activation and p65 levels, causing inflammatory cascades [[18], [19], [20]]. It should be noted that these western blot quantifications are based on a relatively small sample size (N = 4), resulting in high variability and large error bars for p65 measurements. These findings are therefore exploratory, and larger sample sizes will be required in future studies to draw firm mechanistic conclusions. However, B1 could bring these levels back to basal levels observed in the mock condition. This could be due to the interaction of B1 with TDP-43. We have already noted that B1 could bind to the RRM1 domain [4] which is known to interact with the NF-kB and cause its activation [19]. Previously, Pozzi and colleagues [21] showed that targeting RRM1 with an scFv disrupts TDP-43/NF-κB interactions, supporting our mechanistic explanation. The scFv from their team also improved cognitive and motor impairment, but unlike what we did they started treatment in mice already with symptoms onset and used a different mouse model (TDP43 G348C), which may explain the differences in outcomes. These cellular findings align with *in vivo* transcriptomic results. Tg-13R4 mice showed upregulation of multiple immune-related transcripts, including immunoglobulin components (Igkc, Ighg1, Ighg2b, Ighg2c, Ighv3-6, Igkv17-127), complement C4b, and Nlrc5, a negative regulator of NF-κB [22,23]. Such transcriptional signatures indicate persistent immune activation and are consistent with reports from ALS patients and models [[24], [25], [26], [27]]. While part of this signature may be linked to disease-associated stress and inflammatory signalling, it is also important to consider that Tg animals but not WT mice received systemic AAV administration, which may contribute to immune-related transcriptional changes and should therefore be interpreted cautiously. Importantly, comparison of Tg-B1 with Tg-13R4 mice allowed isolation of transcriptional effects associated with scFv B1 treatment while controlling for AAV-related effects. This analysis revealed a distinct transcriptional profile with increased expression of genes linked to stress regulation, immune modulation, and cellular homeostasis (e.g. *Socs3, Ano2),* suggesting that B1 partially attenuates NF-κB–related inflammatory stress. *Socs3*, for example, is a negative regulator of cytokine signalling and NF-κB–associated pathways [28,29], and has been implicated in limiting excessive inflammatory responses in the CNS, supporting the idea that B1 treatment may attenuate inflammatory stress [[30], [31], [32]].

Interestingly, despite these molecular changes, 18-DPA-714 imaging did not detect significant differences between Tg-B1 and Tg-13R4 mice. This is likely because 18F-DPA-714 reflects TSPO expression, which primarily reports microglial activation, and does not capture other inflammatory pathways, including astrocytic, NF-κB–dependent, or T-cell–mediated processes [33,34]. Thus, while B1 clearly modulates immune signalling at the transcriptional level, TSPO-PET imaging may not be suited to detect these effects.

### The scFv B1 induces a partial normalization of disease-associated metabolic disturbances

ALS is also characterized by metabolic alterations that are observed in both animal models and ALS patients [35,36]. Untargeted metabolomic profiling identified specific metabolites that distinguished Tg-B1 mice from Tg-13R4 controls. In the brain, several metabolites altered in Tg-13R4 mice shifted toward WT levels in the B1-treated group, including Hydroquinone and Succinic acid (increased in disease) and Propanoylcarnitine and SN-Glycerol-3-Phosphocholine (decreased in disease). These metabolites are linked to oxidative stress, mitochondrial function, and membrane phospholipid metabolism, processes known to be disrupted in ALS. [[37], [38], [39]]. A similar but weaker pattern emerged in the spinal cord, where only a subset of metabolites showed shifts toward WT levels in Tg-B1 mice. Although modest, these metabolomic adjustments suggest that scFv B1 influences cellular pathways beyond NF-κB signalling, potentially reflecting partial restoration of homeostatic metabolic processes.

Dried blood spots enabled the longitudinal identification of altered metabolites, distinguishing Tg-B1 mice from Tg-13R4 mice. Across the different time points (before injection, 4 months after injection, and before sacrifice), a total of 17 metabolites shifted toward WT levels in the B1-treated group. Most of these metabolites are involved in mitochondrial and energy metabolism, such as lactate, creatinine, L-alanine, and L-glutamate. All these metabolites were decreased in the Tg-13R4 group, consistent with alterations previously described in ALS [[40], [41], [42], [43], [44], [45]].

Markers of oxidative stress and antioxidant defence also shifted toward WT levels following B1 treatment, including Spermidine, Alpha-aminoadipate, and L-Methionine, which have likewise been reported as altered in ALS and other neurodegenerative diseases [[46], [47], [48]].

Furthermore, several gut microbiome–derived metabolites shifted toward WT levels with B1 treatment, including Indole-3-propionic acid, Indoleacrylic acid, and Taurocholic acid. The gut microbiome is now recognized as playing a major role in neurodegenerative diseases, and these metabolites are typically reported as reduced in ALS, contributing to the loss of neuroprotective and anti-inflammatory pathways [[49], [50], [51], [52]].

In addition, certain amino acids and protein turnover markers were identified, such as L-Tryptophan, Thymine, and Deoxyribose. Their alterations in the ALS model may contribute to neurotoxicity and oxidative DNA damage [[53], [54], [55], [56]].

Finally, metabolites involved in vitamin, fatty acid, and organic acid metabolism also shifted toward WT levels following B1 treatment, including D-Pantothenic acid, Lumichrome, 2-Methylbutyrylglycine, and N-Formylglycine [57,58].

These metabolic adjustments suggest that, over time, scFv B1 influences multiple metabolic pathways that are commonly described as altered in ALS.

18-FDG imaging complements these findings by showing persistent regional glucose metabolism alterations in Tg-13R4 mice, including hypermetabolism in cortical and subcortical areas and hypometabolism in the cerebellum. Hypermetabolism in ALS has been linked to glial activation, compensatory neuronal firing, and metabolic stress, whereas hypometabolism often reflects neuronal loss or synaptic dysfunction [36,59]. B1 treatment did not fully restore 18-FDG-PET imaging, but localized metabolic adjustments, such as changes in the hippocampus, suggest some metabolic shifts in oxidative and mitochondrial metabolites.

RNA-sequencing further supports these observations. Tg-13R4 mice showed downregulation of transcripts involved in metabolic and growth-related pathways, including Uox, which plays a role in purine metabolism and oxidative stress regulation in rodents [60], and Pappa2, a modulator of insulin-like growth factor (IGF) bioavailability [36,[61], [62], [63]]. Tg-B1 mice exhibited partial normalization of these transcriptional patterns, alongside reduced expression of several genes involved in neuronal signalling and cellular excitability (Grid2ip, Gbx2, Fzd10, Vipr2, Rgs16, Serinc2) [[64], [65], [66]]. Alterations in metabolic and trophic signalling pathways have been previously implicated in neurodegeneration and ALS pathophysiology and may reflect impaired neuronal support and altered energy homeostasis in the diseased brain [[67], [68], [69]].

This, in addition to metabolite adjustments and localized FDG-PET changes, indicate that B1 acts on both metabolic and trophic pathways, even if global circuit-level metabolic dysfunction remains uncorrected.

### Symptomatic intervention limits functional rescue despite robust CNS expression and target engagement

To model a clinically relevant scenario, treatment was initiated after symptom onset rather than pre-symptomatically, as done in most prior studies. Transgenic mice were already significantly impaired across all behavioural tests at baseline, and longitudinal monitoring did not reveal any improvement in any of the transgenic groups, despite robust CNS AAV transduction and long-term persistence of vector genomes in the brain and spinal cord. This absence of measurable functional benefit likely reflects the reduced therapeutic window available at symptomatic stages, when motor neuron degeneration is already well established and less amenable to reversal advanced disease stage at treatment onset. By the time symptoms appear, motor neuron degeneration, axonal retraction, and glial reactivity are already well established, reducing the therapeutic window for interventions targeting upstream molecular events related to TDP-43. Additionally, the AAV dose, expression level, or anatomical distribution may not have been sufficient to counteract widespread neuronal loss present at treatment initiation. The relatively modest differences in plasma TDP-43 and brain pTDP-43/GFAP between WT and Tg mice reflect well-documented features of this model: TDP-43 accumulation is reported primarily in the spinal cord rather than the brain, and transgenic overexpression introduces inter-animal variability, particularly at intermediate timepoints. Despite these limitations, the neuroinflammatory component in this model is comparable to that observed in mutant SOD1 mice, and our pre-clinical characterization confirmed a robust progressive functional phenotype, supporting its use for therapeutic evaluation.

Assessment of plasma biomarkers NfL and TDP-43 provides further insight. NfL levels were elevated in transgenic mice at baseline and remained stable across all time points, with no differences between scFv-treated and control-treated groups. This stability is consistent with the notion that once neurodegeneration is underway, NfL plateaus and may become insensitive to interventions initiated late in disease [70]. The lack of change in NfL mirrors the absence of functional improvement in behavioural testing, supporting the idea that motor neuron degeneration and axonal loss were already advanced at treatment initiation. Persistently elevated and stable NfL levels may indicate ongoing neuronal degeneration. This suggests that the treatment does not effectively prevent neuronal loss, which continues even after the initial clinical signs have appeared.

In contrast, plasma TDP-43 revealed an intriguing pattern. While levels were comparable across groups at baseline and mid-treatment, WT and control-treated Tg mice exhibited an age-associated increase at the final time point. The convergence of WT and Tg levels at this late timepoint likely reflects a general, genotype-independent rise in circulating TDP-43 associated with aging. Indeed, aging is associated with an accumulation of misfolded or aggregated proteins in the systemic circulation, which may contribute to rising baseline TDP-43 levels over time. Additional contributors may include age-associated alterations in platelet number/activation, as platelets are a known peripheral reservoir of TDP-43 [20,71,72], and possible changes in blood-brain barrier permeability [73]. Notably, this increase was absent in Tg-B1 mice, suggesting a stabilizing effect of B1 on peripheral TDP-43 concentrations. Although this does not necessarily indicate neuroprotection, it does provide evidence of systemic target engagement and supports the possibility that B1 modulates TDP-43 abundance or release from the CNS or peripheral reservoirs. Although B1 clearly engaged aspects of its molecular target, evidenced by reduced NF-κB/p65 activation in cells, modulation of immune-related transcripts *in vivo*, co-localization of scFv B1 with TDP-43 in Tg-B1 brain sections, and the stabilization of plasma TDP-43 levels, the treatment did not affect phosphorylated TDP-43 burden in the brain. This likely reflects the advanced disease stage, in which pTDP-43 inclusions are already hyperphosphorylated, detergent-insoluble, and not readily reversible by upstream interventions. Moreover, B1 does not directly bind to or remodel TDP-43 aggregates; its primary action is the modulation of NF-κB signalling, which is especially prominent in glial cells. As such, reducing glial NF-κB–driven inflammation may lessen ongoing cellular stress but is unlikely to directly influence the clearance of neuronal pTDP-43 inclusions, which depend on proteostatic pathways that are already compromised at symptomatic stages.

Furthermore, scFv B1 expression in mice brain did not modify astroglial activation, pTDP-43 accumulation nor its subcellular distribution at 10 months of age. This suggests that scFv binding was insufficient to alter baseline astrocytic reactivity, promote clearance of pTDP-43 *in vivo* and did not change its intracellular distribution. These findings demonstrate that despite robust expression, this intrabody did not modify TDP-43 pathology *in vivo* at 10 months.

## Conclusion

Together, these multimodal datasets provide a cohesive overall view of B1's therapeutic impact. Inflammatory processes are modulated at the molecular and transcriptional levels, but DPA-PET did not detect global changes, consistent with TSPO's limited coverage of inflammatory pathways. Metabolic and trophic disturbances show partial normalization, whereas network-level dysfunction persists. Plasma biomarkers further indicate target engagement through stabilization of circulating TDP-43, while stable NfL and persistent functional deficits reflect ongoing neurodegeneration.

Overall, the data suggest that B1 modulates key upstream mechanisms of TDP-43 proteinopathy, including NF-κB activation, immune signalling, and metabolic stress. However, in our experimental paradigm, intervention at a symptomatic stage is insufficient to reverse advanced neuronal loss or restore motor function. Limitations such as late treatment timing, suboptimal vector coverage, and the inherent irreversibility of established TDP-43 pathology likely contributed to these outcomes.

Collectively, and while definitive proof of direct binary interaction in intact brain tissue remains technically challenging, the convergence of cellular, molecular, transcriptomic, and *in situ* evidence presented here constitutes the strongest demonstration yet that scFv B1 engages TDP-43 within the CNS following AAV-mediated delivery, extending our previous report [4].

Given the known influence of sex on ALS progression and neuroinflammation [[74], [75], [76], [77]], future studies should include male subjects and additional TDP-43 models to assess the generalizability of these findings.

Looking forward, earlier intervention, optimized vector design (e.g., AAV serotype selection, and promoter choice to target neurons and/or glia), higher AAV doses, and testing in additional TDP-43–driven animal models may enhance understanding of B1's full potential. Combination strategies that pair TDP-43–modulating approaches with neuroprotective or network-supporting therapies could further improve outcomes. These refinements will help clarify B1's full potential and guide future translational efforts in ALS.

## Author contributions

Y.O., and H.B. contributed to the conception of the study, Y.O., A.D., M.P., D.L., R.H., A.C., and S.M. performed experiments and analyzed the data, L.G. and S.S. performed PET experiments and analyzed the data, C.D. performed metabolomics experiments, P.M. performed screening of the scFv, T.A. analyzed RNA-seq data, B.S. supplied the AAV, F.M. and F.T. performed and analyzed phenotypic characterization on the animal model, C.V., C.A., P.C., and P.V., reviewed and validated the study, H.B. supervised the study, Y.O. and A.D. wrote the manuscripts with inputs from all the authors.

## Studies in animals

Our studies were carried out in accordance with Guidance on the operation of the Animals (Scientific Procedures) Act 1986 and EU Directive 2010/63 for the protection of animals used for scientific purposes.

## Funding Information

This work was supported by the 10.13039/501100006510ARSLA (Association pour la Recherche sur la Sclérose Latérale Amyotrophique), the 10.13039/501100015760INSERM, the LabEx MabImprove program, and the ARD2020 program of the Région Centre-Val de Loire.

## Declaration of interests

The authors declare no conflict of interests.

## Figures and Tables

**Supplementary Fig. 1:**
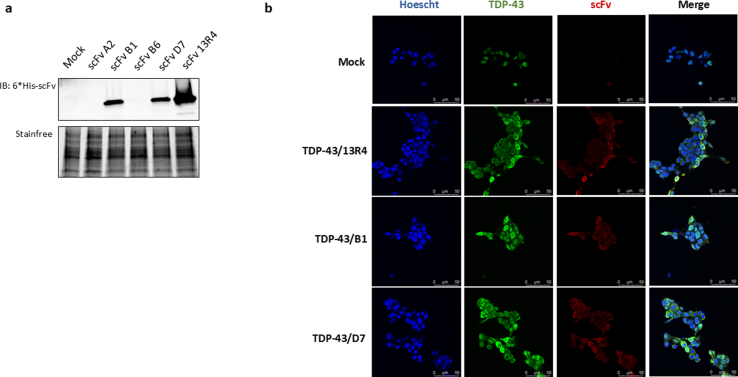
**B1 is well expressed in NSC-34. a)** Stainfree gel and immunoblot of 6∗his-tagged scFv in NSC34 total lysates after 48 h of overexpression. Mock: transfection of empty vector. **b)** Immunofluorescence detection of TDP-43 and scFv-6∗His in NSC34 cells. Nuclei are counterstained with Hoescht (blue), TDP-43 is shown in green, and the scFv is shown in red (scale bar = 50 μm).

**Supplementary Fig. 2:**
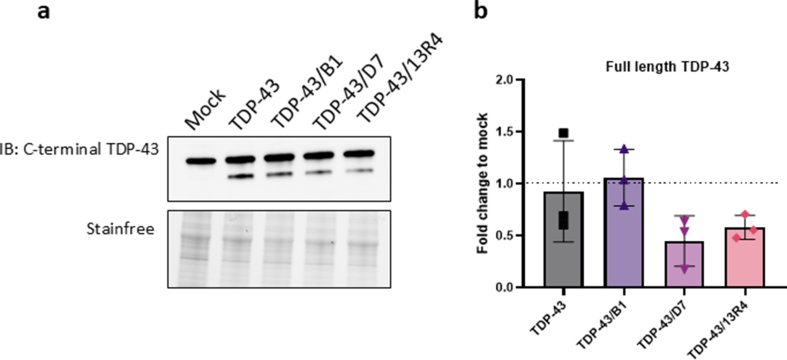
**B1 has no effect on TDP-43 in NSC-34. a)** Stainfree gel and immunoblot of C-terminal TDP-43 in NSC34 total lysates after 48 h of overexpression. Mock: transfection of empty vector. TDP-43: transfection of TDP-43 and empty vector. TDP/B1, TDP/D7, TDP/Control: co-transfection of TDP-43 and corresponding intrabody. **b)** Quantification of full-length TDP-43 in total lysate normalized to stain-free lanes. Values were normalized to the mock condition (dotted line). Mann-Whitney test; N = 4.

**Supplementary Fig. 3:**
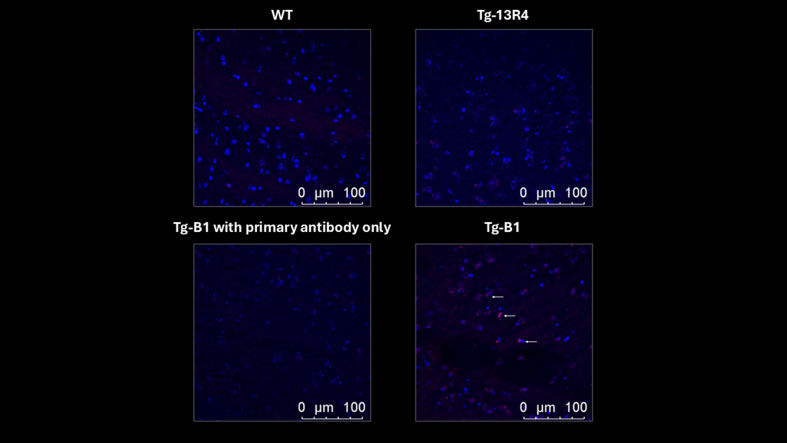
***In vivo* proximity ligation assay (PLA) demonstrates sub-40 nm interaction between intrabody B1 and TDP-43 in mouse brain.** PLA was performed on brain sections from mice sacrificed 6 months post-treatment to detect endogenous TDP-43 in close proximity (<40 nm) to intrabody B1. Four conditions were included: wild-type (WT) untreated mice; Tg-13R4 (negative specificity control); Tg-B1 with TDP-43 antibody only (omitting 6∗His antibody); and transgenic mice treated with intrabody B1 with both PLA PLUS and MINUS probes (B1/TDP-43 PLA). White arrows indicate PLA puncta, indicating B1 and TDP-43 to be in proximity (<40 nm). Scale bar = 100 μm.

**Supplementary Fig. 4:**
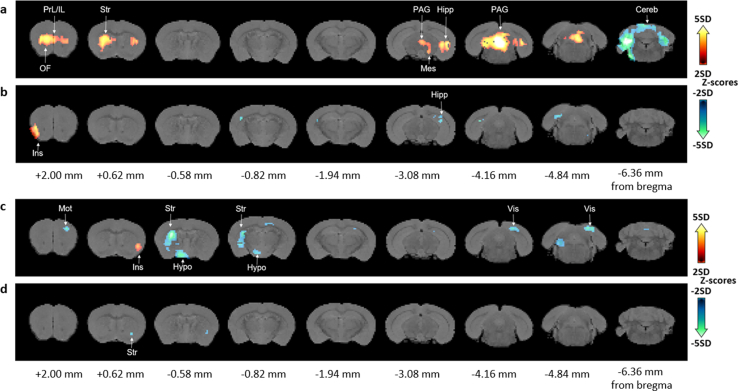
**a-b) Molecular PET imaging highlighting differences in cerebral metabolic activity**, assessed by [ˆ18F]FDG uptake, between (A) Tg-13R4 and WT mice, and (B) Tg-B1 and Tg-13R4 mice. **c-d) Molecular PET imaging highlighting differences in neuroinflammation**, assessed by [ˆ18F]DPA-714 uptake, between (C) Tg-13R4 and WT mice, and (D) Tg-B1 and Tg-13R4 mice. Significant changes in tracer uptake are shown on z-score maps fused with an MRI template (Mirrione template). Regions with significantly increased tracer uptake are represented in red to yellow, whereas regions with significantly decreased uptake are shown in blue to white. Abbreviations: Cereb, cerebellum; Hipp, hippocampus; Hypo, hypothalamus; IL, infralimbic cortex; Ins, insula; Mot, motor cortex; OF, orbitofrontal cortex; PAG, periaqueductal grey; PrL, prelimbic cortex; Mes, mesencephalon; Str, striatum; Hypo, Hypothalamus; Mot, Motor cortex; Mes, Mesencephalon Vis, Visual cortex.
